# Emerging complications of COVID-19 in a subset of Indian population: a pathological review with clinico-radiological case scenarios

**DOI:** 10.1186/s43055-021-00680-1

**Published:** 2022-02-15

**Authors:** Aniket Agarwal, Prachi, Andaleeb Haider, Ekansh Lalit, Ajay Kumar Agarwal, Seema Agarwal

**Affiliations:** 1 Department of Radiology, ABVIMS and RML Hospital, New Delhi, India; 2 Department of Pathology, Dharamshila Narayana Superspeciality Hospital, New Delhi, India; 3 Department of Radiology, ABVIMS and RML Hospital, New Delhi, India; 4 Department of Ophthalmology, Narayana Nethralaya, Bangalore, Karnataka India; 5 Agarwal Polyclinic and Laboratory, New Delhi, India

**Keywords:** COVID-19, Pathogenesis of COVID-19, Cytokine storm, Systemic complications of COVID-19

## Abstract

**Background:**

Coronavirus disease 2019 (COVID-19) caused by severe acute respiratory syndrome coronavirus 2 (SARS-CoV-2), which was declared a pandemic by the World Health Organization on 11 March 2020 has been reported in most countries around the world since its origins in Wuhan, China. As of September 2021, there have been over 229 million cases of COVID-19 reported worldwide, with over 4.7 million COVID-19–associated deaths.

**Body:**

The devastating second wave of the COVID-19 pandemic in India has seen a rise in various extrapulmonary manifestations. One of key components in the pathogenesis of COVID-19 is downregulation of ACE-2, which is expressed on many organs and counterbalances the pro-inflammatory effects of ACE/angiotensin-II axis. This leads to influx of inflammatory cells into alveoli, increased vascular permeability and activation of prothrombotic mediators. Imaging findings such as ground glass opacities, interlobular septal thickening, vascular dilatation and pulmonary thrombosis correlate well with the pathogenesis.

**Conclusion:**

We hypothesize that the systemic complications of COVID-19 are caused by either direct viral invasion or effect of cytokine storm leading to inflammation and thrombosis or a combination of both. Gaining insights into pathobiology of SARS-CoV-2 will help understanding the various multisystemic manifestations of COVID-19. To date, only a few articles have been published that comprehensively describe the pathophysiology of COVID-19 along with its various multisystemic imaging manifestations.

## Background

Coronavirus disease 2019 (COVID-19) is an infectious disease caused by severe acute respiratory syndrome coronavirus 2 (SARS-CoV-2), which is an enveloped beta coronavirus [1]. COVID-19 had its origins in Wuhan, China in December 2019 which spread worldwide resulting in lockdowns and restrictions. World Health Organization (WHO) had officially declared COVID-19 as a pandemic on 11 March 2020.

The binding and entry into the host cell is mediated by its surface protein S (the spike protein) that recognizes the angiotensin converting enzyme 2 (ACE-2) receptors present on epithelial surfaces of the lungs, heart, kidney, and intestines, followed by biosynthesis, maturation and release of new virus particles. COVID-19 follows a biphasic pattern of illness which constitutes an early viral response and a late inflammatory phase [2].

Most patients experience a mild flu-like illness and have favourable prognosis, however the elderly and immunocompromised are more predisposed to severe and critical illness [3]. In patients with severe illness, SARS-CoV-2 elicits an aberrant response which may quickly progress to severe pneumonia and acute respiratory distress syndrome (ARDS) with or without other end organ failures [4].

## Main text

In this article, we provide an insight into the pathophysiology and radiological appearances of few multisystemic manifestations of COVID-19, which are becoming more commonly recognized with increasing case load and use of imaging.

We conducted a literature search in Pubmed, Scopus and Google Scholar for articles relevant to pathogenesis of SARS-Cov-2 and searched for terms coronavirus, severe acute respiratory syndrome coronavirus 2, SARS-CoV-2, cytokine storm, clinical complications related to different organ systems from January 2020 to March 2021. The articles closely related to our study were included in this review and have been mentioned in references as they appear in the discussion.

### Virology

SARS-CoV-2 is an enveloped single stranded ribonucleic acid (RNA) beta coronavirus. Its genome shows 80% similarity to SARS-CoV-1 and 96% similarity to bat coronavirus RaTG_13_ [1]. Out of six types of coronaviruses that have been identified to cause human disease, SARS-CoV-1 and Middle East respiratory syndrome (MERS) have already resulted in pandemics. SARS-CoV-2 is more infectious than SARS-CoV-1 because of structural differences in its surface proteins that enables stronger binding to ACE-2 receptors and greater affinity for upper respiratory tract and conjunctiva making it more efficient at invading the host cell [5–7].

### Epidemiology

Since the first reports of cases from Wuhan, a city in the Hubei Province of China, at the end of 2019, the disease has spread like a wildfire in more than 200 countries globally involving all continents barring Antarctica. More than 229 million cases have been reported worldwide as of 21 September 2021 with more than 4.7 million deaths. India accounts for more than 33 million reported cases and 445,000 deaths [8].

It has been reported that the official counts represent only the tip of the iceberg underestimating the overall burden of COVID-19, as only a fraction of acute infections are diagnosed and reported. Seroprevalence surveys in the United States and Europe have revealed that the rate of prior exposure to SARS-CoV-2, as reflected by seropositivity, exceeds the incidence of reported cases by approximately tenfold or more [9, 10].

The primary mode of transmission is via respiratory droplets with infection occurring when these particles are inhaled or deposited on nasal, conjunctival or oral mucous membranes [7].

Most transmission occurs through close range and more duration of contact especially 15 min face to face and within 2 m [11]. Transmission via aerosol in poorly ventilated settings is also known to play an important role, while extent of fomite transmission and role of fecal shedding are not fully understood [2, 12].

### Diagnosis

Real-time reverse transcriptase polymerase chain reaction (RTPCR) test from nasopharyngeal and oropharyngeal swabs is considered the gold standard for diagnosis, however recent variants like the delta variant are capable of evading the test rendering it false negative. False negative results may occur with any molecular test for the detection of SARS-CoV-2 if a mutation occurs in the part of the virus’ genome assessed by that test [13].

Other causes of false negative RTPCR test are mutations in the primer and probe target regions in SARS-CoV-2 genome, viral load kinetics and faulty sampling procedures. Thus, a negative RTPCR should not be used as the sole criterion for treatment and management decisions in suspected cases of COVID-19, in presence of strong clinical suspicion and prevailing epidemiology of COVID-19 [14]. The WHO recommends the use of chest imaging in cases where RTPCR is not available or has delayed results or when there is high clinical suspicion despite negative RTPCR [15].

### Pathophysiology

**Fig. 1:**
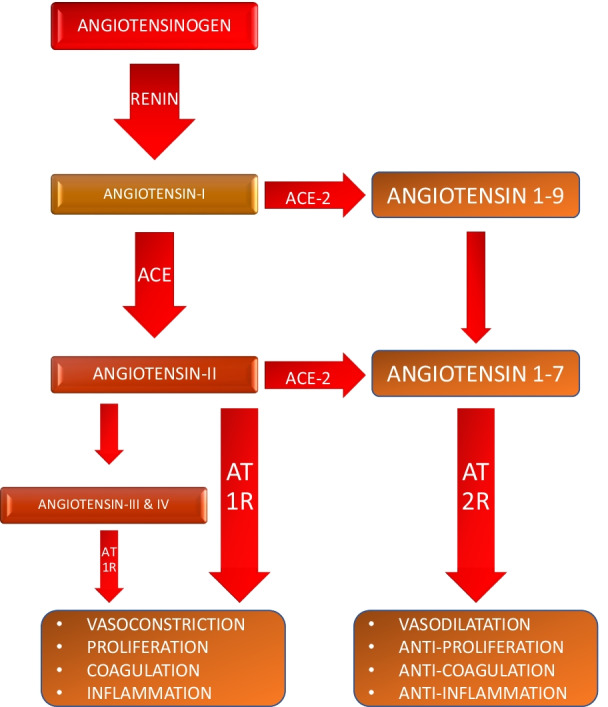
The renin angiotensin system. ACE: Angiotensin converting enzyme; ACE-2: Angiotensin converting enzyme-2; AT1R/AT2R: Angiotensin I receptor/Angiotensin II receptor; Angiotensin 1–7 and 1–9: shorter peptides of angiotensin

**Fig. 2:**
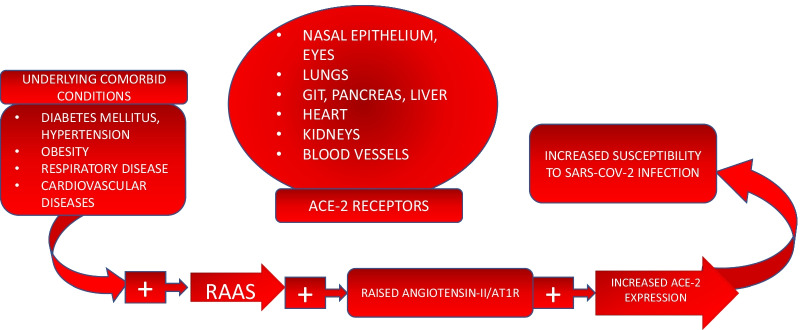
Distribution of ACE-2 receptors and underlying co-morbid conditions

**Fig. 3:**
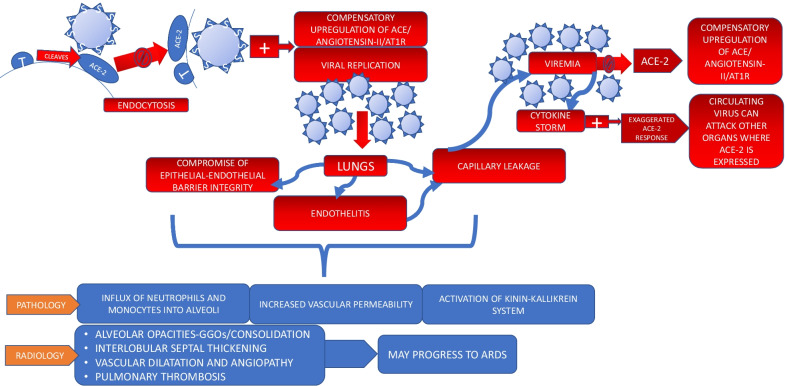
Pathogenesis of COVID-19. T: TMPRSS2 (transmembrane serine protease 2)

The Renin-angiotensin-system along with the mechanism of SARS-CoV-2 invasion into host cells and the ensuing effects has been briefed in Figs. 1, 2 and 3.

The spike protein, present on cell surface of SARS-CoV-2 contains receptor binding domain that attaches to ACE-2 receptor present on nasal epithelium, eyes, lungs, gastrointestinal tract, liver, pancreas, heart and blood vessels. TMPRSS2 (transmembrane serine protease 2) or furin (enzyme) primes the S protein and also cleaves the ACE-2 receptor causing downregulation of ACE-2. This mediates invasion of the virus into the host cell which is then followed by biosynthesis, maturation and release of new particles [16] (Fig. 3).

One of key components to understanding the pathogenesis of COVID-19 is ACE-2, which is a negative regulator of renin angiotensinogen aldosterone system (RAAS) and counterbalances the pro-inflammatory effects of angiotensin-II/AT1R receptor axis by breaking down angiotensin-II to shorter peptides namely angiotensin 1–9 and angiotensin 1–7 (Fig. 1). As the viral replication occurs, ACE-II downregulates, thus inhibiting breakdown of angiotensin-II thereby causing hypokalemia, vasoconstriction and ARDS like features [17–22].

In parallel to viremia which occurs with capillary damage, there is surge in inflammatory mediators leading to cytokine storm which leads to overexpression of ACE-2 response so as to counterbalance the ACE/angiotensin-II axis mediated inflammatory effects. Given the fact that ACE-2 receptors are expressed on most human organs, which is now exaggerated because of the cytokine storm, the circulating viruses use this window to attack these organs (Fig. 3). This vicious cycle then continues leading to systemic failure [23].

The cytokine release syndrome (CRS) or cytokine storm is the uncontrolled systemic inflammatory response that occurs due to immunological misfiring that causes release of high amounts of proinflammatory cytokines along with complement components and coagulation dysfunction [23, 24].

We hypothesize that various multisystemic complications caused by SARS-CoV-2 can be explained by direct viral attack (by means of ACE-2 receptors), or immune cell mediated cytokine storm related pro-inflammatory signals along with upregulation of ACE/angiotensin-II axis (due to downregulation of ACE-2) or a combination of both. This may cause myriad of systemic complications.

**Fig. 4:**
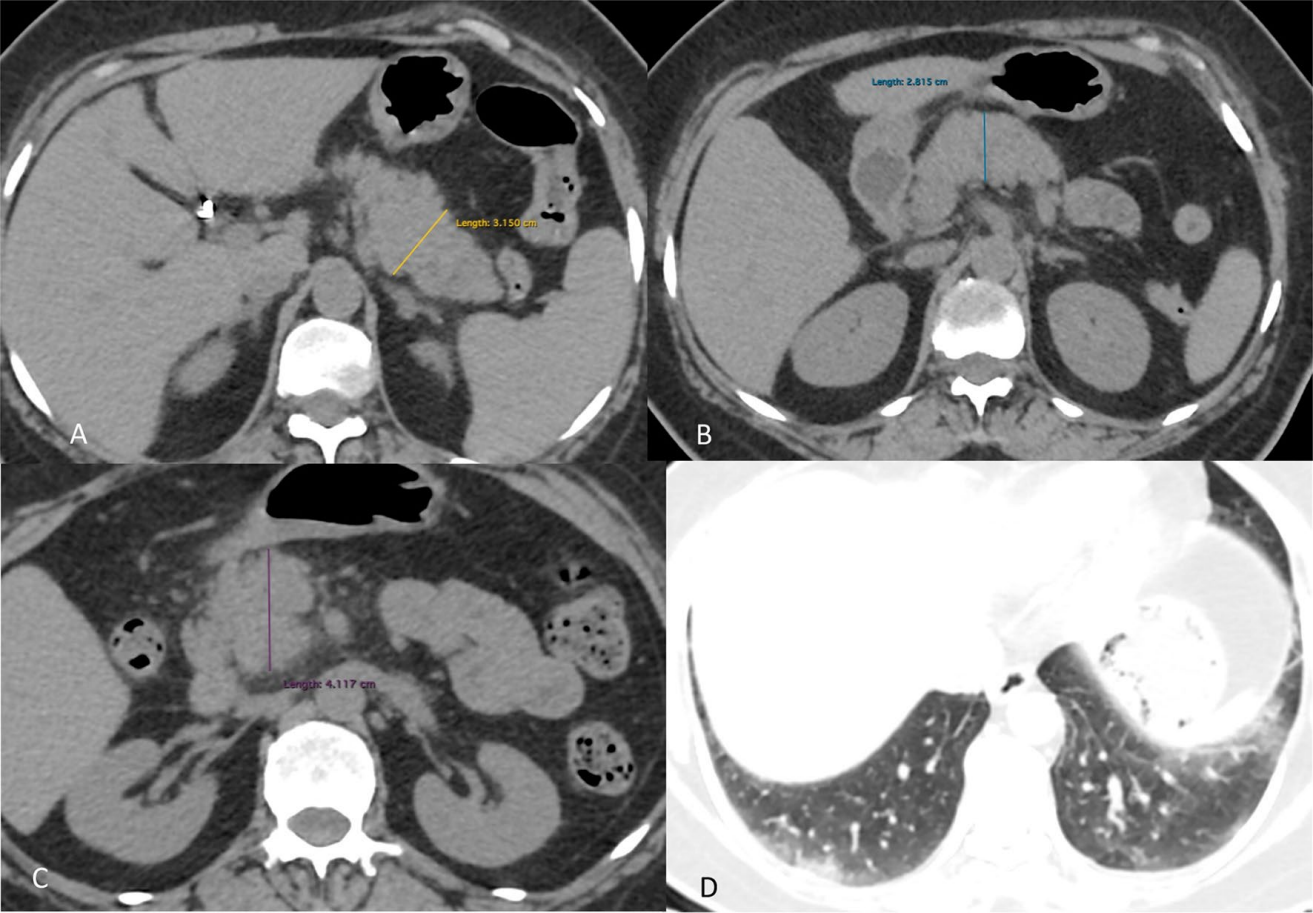
Non contrast CT images (**A**–**C**) reveal bulky pancreas with peripancreatic fat stranding. No free fluid is seen. Evidence of post cholecystectomy clips is noted in image **A**. **D** Lung window of the same patient shows COVID-19 changes in both lung fields

Pancreatic islet cells and exocrine cells of pancreas express ACE-2 receptors. Acute pancreatitis may be idiopathic or caused by several etiological factors such as gall stones, alcohol abuse, metabolic disorders, autoimmune diseases, drugs, toxins and viruses such as mumps, Coxsackie B, measles, Epstein–Barr and hepatitis A, B and E. Association between H1N1 influenza and acute pancreatitis has also been reported [25, 26]. One study has also suggested a direct impact of COVID-19 infection on the pancreas [27]. There were  no reasons for clinical suspicion or direct evidence of above-mentioned etiological factors in our case presented later (Case 1, Fig. 4). The best possible diagnosis was COVID-19 induced pancreatitis.

Evidence of raised serum amylase and lipase should not be directly attributed to acute pancreatitis as many patients with COVID-19 who present with gastrointestinal symptoms may show elevated pancreatic enzymes [28]. Hence, imaging is necessary to rule out acute pancreatitis. Ultrasound is the initial imaging modality of choice. In our case, ultrasound (done outside) did not reveal any abnormality. Therefore, non-contrast CT scan of abdomen was done in view of raised serum creatinine. Contrast enhanced computed tomography provides over 90% sensitivity and specificity for the diagnosis of acute pancreatitis [29].

The current second wave of the COVID-19 pandemic in India has seen a rise in the rhino-orbital mucormycosis co-infections in COVID-19 patients.

Fungal sinusitis is broadly classified as invasive and non-invasive. Invasive fungal sinusitis consists of fungal hyphae within the mucosa, submucosa, bone, or blood vessels of the paranasal sinuses [30].

Mucormycosis is an angioinvasive disease caused by fungi of the order Mucorales such as *Rhizopus*, *Mucor*, *Rhizomucor*, *Cunninghamella* and *Absidia*. The prevalence of mucormycosis in India is approximately 0.14 cases per 1000 population, about 80 times the prevalence in developed countries [31]. Underlying immunosuppressive states such as diabetes, hematological malignancies, organ transplantations, treatment with immunosuppressants are common predisposing factors. COVID-19 has been associated with increasing trend in fungal infections. Complications of orbital and cerebral involvement are more frequent in diabetic ketoacidosis and with concomitant use of steroids [32]. Prolonged use of corticosteroids at a therapeutic dose of ≥ 0.3 mg/kg for at least three weeks in the past 60 days is considered a risk factor for invasive fungal diseases [33]. Further, IL-6-inhibiting drugs such as tocilizumab, used for mitigation of cytokine storm, may cause immune dysregulation thereby increasing the risk of secondary infections, without much clinical benefit in patients with COVID-19 [34, 35]. COVID-19 with acute respiratory distress syndrome may also predispose patients to secondary infections as a result of immune dysregulation [36]. SARS-CoV-2 directly impairs cell-mediated immune response, by virtue of reduced levels of circulating lymphocytes and T cell subsets [37].

Rhino-orbito-cerebral mucormycosis is considered as the most common manifestation of mucormycosis that is thought to be acquired via the inhalation of fungal spores into the paranasal sinuses. The spores invade the nasal mucosa and form angio-invasive hyphae that cause infarction of involved tissue which manifests as black palatal or gingival eschars. It may lead to maxillary and subsequent orbital spread via ethmoidal sinus. Perforation of the nasal septum may also be seen. It usually presents with an acute onset of fever, facial pain, nasal congestion, headache, perinasal swelling, facial numbness, and visual changes such as diplopia and proptosis [38].

The multiplanar capabilities of MRI with its superior soft tissue depiction make it a valuable modality that is used not merely in the diagnosis of mucormycosis, but also in delineating the anatomical extent of disease as well as its complications [32, 39]. Bony changes are better assessed by CT.

Both MRI and non-contrast CT demonstrate mucosal thickening and/or soft-tissue within the lumen of the involved paranasal sinus and nasal cavity. The soft tissue contents show variable signal intensity on T1- and T2-weighted images. Hypertrophy of nasal turbinates is seen with nasal involvement. Post-contrast enhancement can be seen in the thickened mucosa and involved tissues. However, contiguous areas of non-enhancing soft tissue may be seen within the necrosed turbinates and/or paranasal sinuses, known as the “black turbinate sign”. The infarcted mucosa may show restriction of diffusion [40]. In general, sinusitis demonstrates enhancement of the peripheral mucosa on T1 weighted post contrast sequence. Lack of enhancement of the mucosa is typical of rhinocerebral mucormycosis because the hyphae invade smaller vessels supplying the mucosa [40].

Severe unilateral nasal cavity soft-tissue thickening is the most consistent, though nonspecific, feature [40]. Mild fat stranding in pterygopalatine fossa, peri-antral, malar, masticator spaces with even subtle findings in sinuses is suggestive of invasive fungal sinusitis in appropriate clinical setting. More extensive changes such as retroantral fat pad inflammation, bone erosion, and orbital or intracranial invasion are more specific but late features [41].

In contrast, allergic fungal sinusitis shows bilateral asymmetrical involvement, usually pansinusitis causing expansion of sinuses, with hyperdense fungal contents on CT due to allergic mucin. There is typical low signal intensity or signal void on T2W images due to high concentration of metals like iron, magnesium, manganese concentrated by the fungal organisms along with high protein and low free water content of the allergic mucin [41].

Aggressive bone destruction of the sinus walls occurs rapidly with intracranial and intraorbital extension [41]. Sometimes, bone erosion and mucosal thickening may be subtle. Extension beyond the sinuses may occur with intact bony walls as they tend to spread along vessel walls. Facial numbness, caused by fifth cranial nerve involvement, indicates that the infection has spread beyond the sinuses. Orbital involvement occurs in the form of inflammatory changes in the orbital fat and extraocular muscles and resultant proptosis. Obliteration of the peri-antral fat is a subtle sign of such extension [41]. Intracranial invasion occurs in the form of leptomeningeal enhancement in early infection and cerebritis, granulomas, and cerebral abscess formation may be encountered in advanced stages. Intracranial granulomas appear hypointense on T1- and T2-weighted images with minimal enhancement on post contrast images [41]. Spread to the brain may occur via the orbital apex, orbital vessels or via the cribriform plate causing fungal abscess, cranial nerve palsy, cavernous sinus thrombosis, carotid artery involvement and stroke [42].

**Fig. 5:**
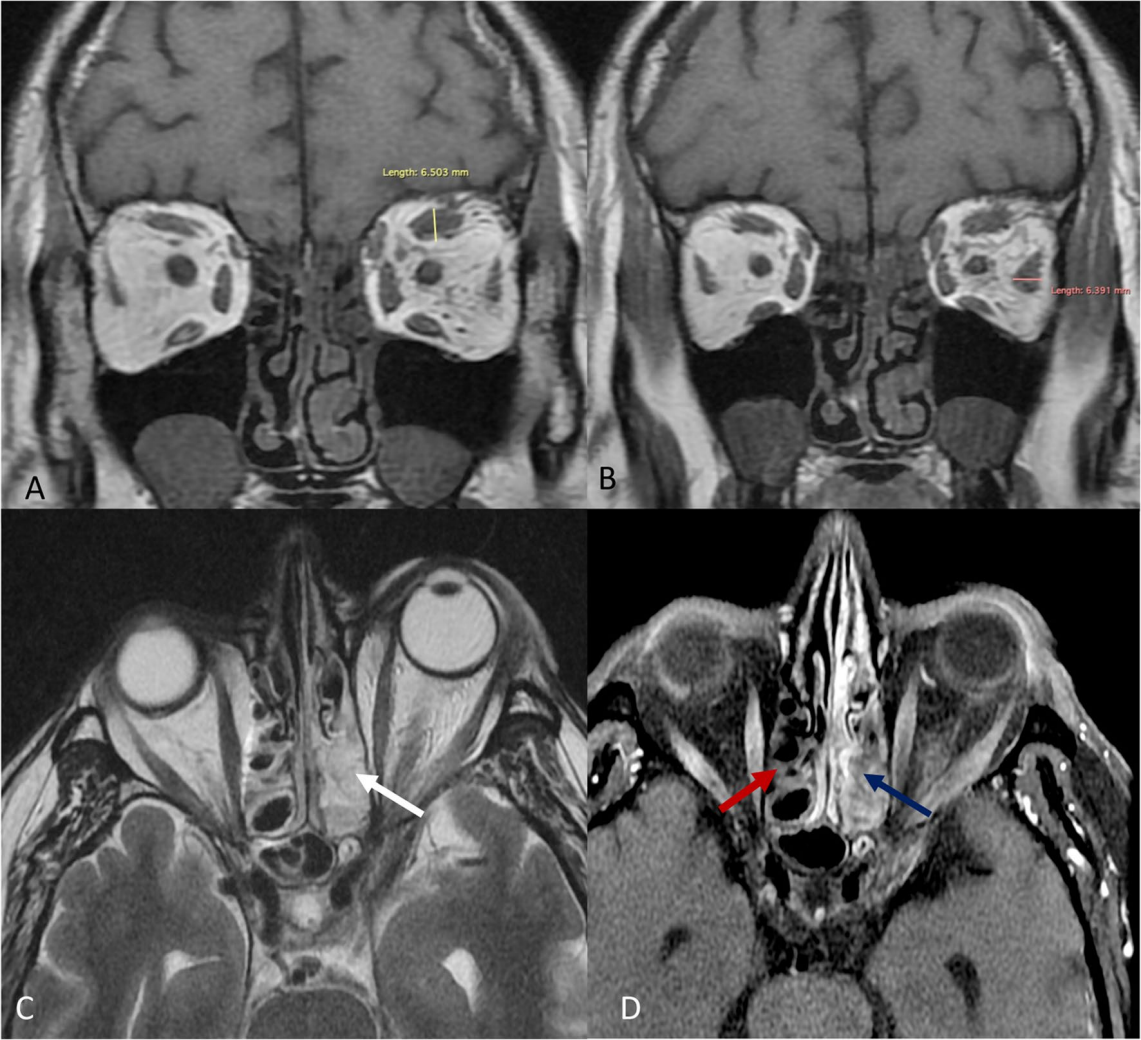
T1W coronal images (**A**, **B**) show bulky superior rectus, lateral rectus and superior oblique muscles and intraconal fat stranding along with bulky middle and inferior turbinates. Polypoidal mucosal thickening is also seen in both maxillary sinuses. **C** T2W axial image shows proptosis of left orbit with preseptal swelling along with mucosal hypertrophy and T2 hyperintense contents in ethmoid sinus predominantly on left side (white arrow). **D** T1 post contrast axial image shows contiguous non-enhancing areas involving the walls of ethmoid sinus and middle turbinate on left side- black turbinate sign (blue arrow). Walls of right ethmoid sinus show lack of enhancement (red arrow)

**Fig. 6:**
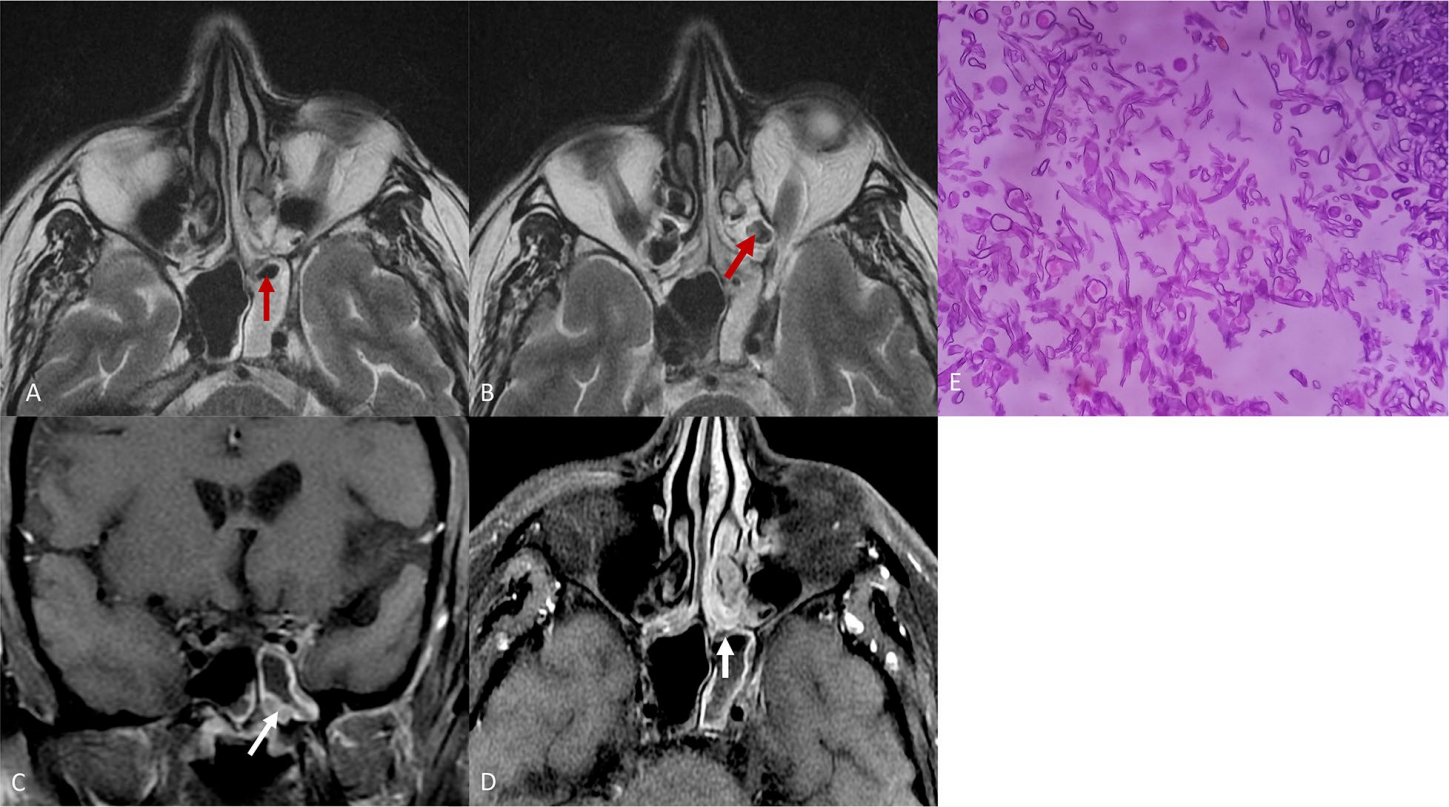
(same patient as in Fig. 5: **A**, **B**: T2W axial images showing T2 hyperintense contents in ethmoid and sphenoid sinuses on left side with areas of signal void  (red arrows) suggestive of fungal contents within. T1 post contrast coronal (**C**) and axial (**D**) images show patchy non-enhancing walls of sphenoid sinus on left side (white arrows) with normal enhancement of adjacent walls. **E** Microphotograph from debrided material showing magenta coloured aseptate fungal hyphae seen in the necrotic material likely to be mucormycosis (PAS stain X400)

Rhino-orbital mucormycosis may occur even in patients who have been recently discharged after recovering from COVID-19 as seen in case number 2 illustrated below in Figs. 5 and 6.

A common fungal infection that can cause secondary pulmonary infection in severely immunocompromised patients is aspergillosis, which is caused by *Aspergillus fumigatus*, an opportunistic fungal pathogen. Recent studies reveal occurrence of aspergillosis in 20–30% of the severely ill or ventilated COVID-19 patients, hence establishing an association between COVID-19 and pulmonary fungal infections, which is referred to as COVID-19 Associated Pulmonary aspergillosis (CAPA) [43]. CAPA's risk factors are similar to those of severe COVID-19 [44]. It has been shown that hospitalized COVID-19 patients who develop ARDS become more susceptible to acquire superimposed infections caused by bacteria and *Aspergillus* species, which is associated with high mortality rates and may prolong the acute phase of COVID-19 [45].

In view of the high morbidity and mortality, early diagnosis and treatment are imperative. A combination of HRCT chest and Aspergillus antigen tests on bronchoalveolar lavage (BAL) and serum, including galactomannan enzyme-linked immunosorbent assay or lateral flow tests, or Aspergillus PCR are required for diagnosis of CAPA. As BAL is an aerosol generating procedure, an alternative is the beta-D-glucan [46].

**Fig. 7:**
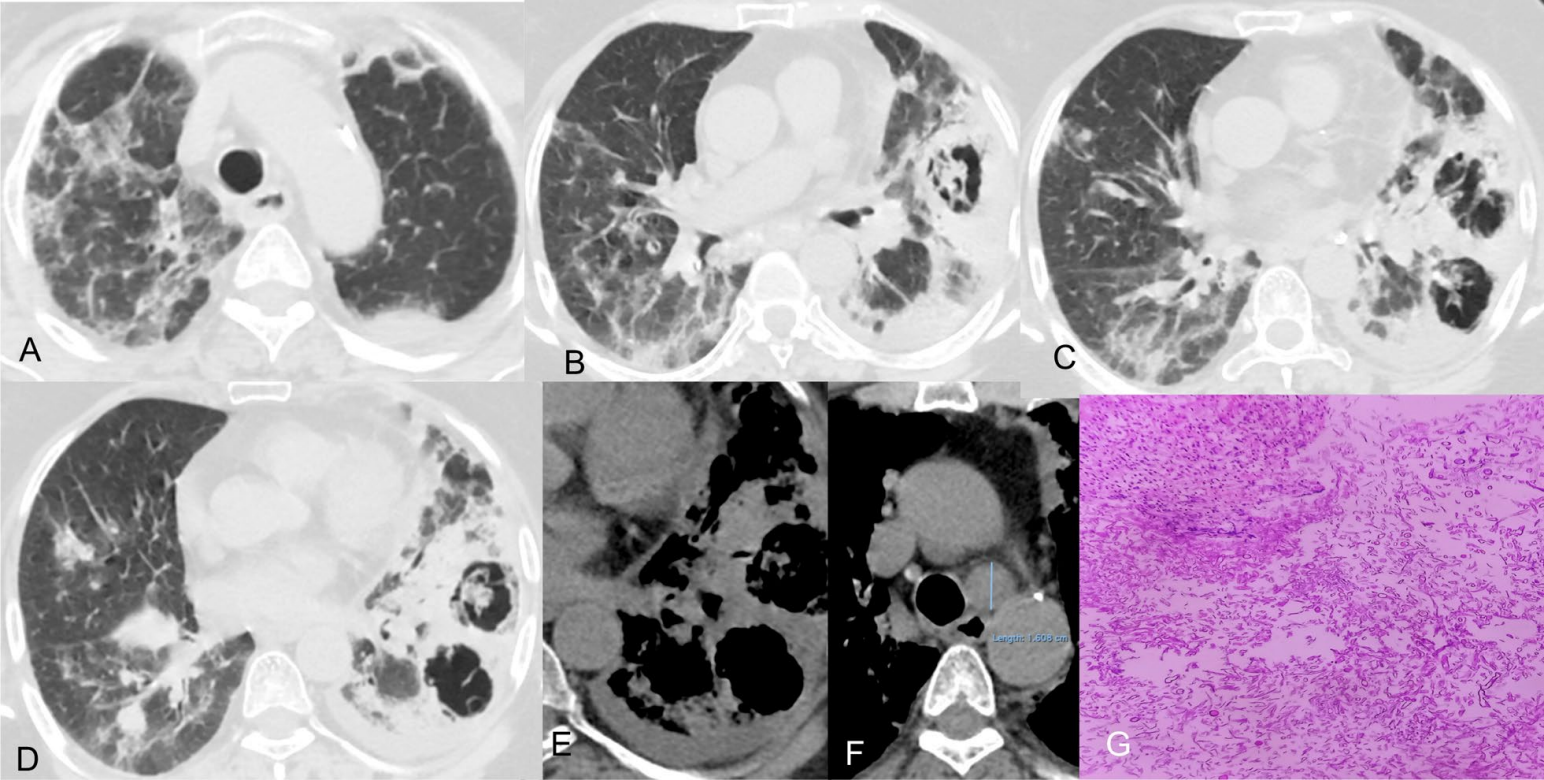
HRCT chest lung window. (**A**–**D**) Two large thick walled cavities are seen in left lower lobe with internal soft tissue density contents giving bird’s nest appearance on the background of changes of COVID-19 pneumonia. **E**, **F** Mediastinal window images showing soft tissue density contents within cavity and left pleural effusion along with an enlarged aorto-pulmonary node (**F**). **G** Microphotograph from lung biopsy specimen showing magenta coloured septate fungal hyphae in the necrotic material likely to be mucormycosis (H&E stain X400)

The reverse halo sign refers to the peripheral consolidation surrounding a central area of ground-glass opacity. Associated irregular and intersecting areas of stranding or irregular lines may be present within the area of ground-glass opacity or a cavity which along with surrounding consolidation is referred to as the bird’s nest sign (Fig. 7). These signs are suggestive of invasive fungal infection (e.g., angioinvasive Aspergillus infection or mucormycosis) in susceptible patient populations [47].

COVID-19, being a pro-inflammatory and pro-thrombotic condition as detailed above (Fig. 1 and 3), produces a state of hypercoagulability thereby leading to clot formation in vessels. Predisposing vascular risk factors such as diabetes, hyperlipidemia, smoking may further exacerbate the risk. In milder cases of COVID-19, direct viral invasion leading to endothelitis is thought to be the causative factor, whereas in individuals with severe COVID-19, the pro-inflammatory cytokine storm increases the chances of pre-existing plaque rupture and subsequent thrombus formation [48].

The article has been enriched with few interesting multisystemic post COVID complications.

*Case 1* A 52-year-old female, recently recovered from RTPCR proven COVID-19, presented with nausea, vomiting and acute upper abdominal pain radiating to back for the last 2 days. There was history of mild diarrhea during initial phase of COVID-19 along with mild upper respiratory tract symptoms. Vaccination status was negative. Laboratory investigations revealed raised serum amylase and lipase (910 U/L and 652 U/L respectively); normal liver profile; raised total leukocyte count (19,300/mm^3^); raised CRP (30 mg/L) and raised serum creatinine (1.7 mg/dL). The lipid profile was normal.

The physical examination revealed nondistended, soft abdomen with epigastric tenderness. No mass was palpable. Rest of the general physical examination was unremarkable with no signs of dehydration or jaundice. There was past history of cholecystectomy 14 years back due to gall stones, however, without any history of biliary calculi or pancreatitis. The patient did not undergo any invasive procedure, namely endoscopic retrograde cholangiopancreatography or recent surgery. No history of trauma, or recent hospitalization was present. The patient was non-alcoholic.

Ultrasound was unremarkable. Non-contrast CT abdomen revealed enlarged pancreas with peripancreatic fat stranding with no evidence of peripancreatic fluid collection or ascites (Fig. 4a–c).

Limited sections of thorax revealed multifocal patchy subpleural ground glass opacities in both lower lobes (Fig. 4d). The patient was treated conservatively and recovered well.

*Case 2* A 54-year-old non-hypertensive male with uncontrolled diabetes, recently recovered from RTPCR positive moderate COVID-19, who was discharged 10 days back after being admitted in hospital for 7 days, presented with headache, swelling around left eye with painful restriction of eye movements and blurring of vision for the last 3 days. The right eye was unaffected. On general physical examination, the patient was febrile with normal vitals and oxygen saturation of 96%. During hospital stay, he had received intravenous methylprednisolone 40 mg BD which was tapered gradually.

On ophthalmological examination, mild periocular edema and proptosis were seen. Best corrected visual acuity was 6/6 in right eye and 6/12 in left eye. Left eye abduction was severely limited.

*Intraocular pressure* 16 mm Hg in right eye, 20 mm Hg in left eye.

*Ocular motility of left eye* abduction: − 2, elevation: − 2.

*Pupils* round, regular, reacting to light, no relative afferent pupillary defect. Confrontation of visual field was full.

*Lid* edema.

*Slit lamp examination* Conjunctival chemosis was present in left eye. Cornea, anterior chamber, iris and lens were normal.

Fundoscopy was unremarkable.

*Laboratory parameters* CRP: 55 mg/L; D-dimer: 913 ng/ml; serum ferritin: 703 ng/ml. TLC was elevated (14,500/mm^3^). Rest of the parameters (LFT, KFT, thyroid profile) were within normal limits.

CEMRI of paranasal sinuses and orbits revealed evidence of rhino-orbital mucormycosis as shown in Figs. 5 and 6.

*Case 3* A 71-year-old female patient with uncontrolled diabetes mellitus and hypertension, was admitted with severe COVID-19 one month back. The patient recovered well and was discharged from hospital after a stay of 10 days. She received methylprednisolone 80 mg IV BD for 10 days. She was prescribed dexamethasone 6 mg BD for 3 days followed by 6 mg OD × 3 days on discharge among other medicines. Now the patient complaints of fever and cough of 3 days duration, 20 days after dis- charge from hospital. Repeat RTPCR was negative.

HRCT was done for further evaluation which revealed bird nest sign suggestive of mucormycosis, on the background of COVID-19 as shown in Fig. 7.

*Case 4* An 80-year-old non-hypertensive non-diabetic male admitted with severe COVID-19 complained of sudden onset of severe headache, loss of vision in left eye, periorbital swelling with painful restriction of eye movement, nasal stuffiness and discharge with mild epistaxis, facial pain and swelling. The patient has history of chronic kidney disease for 3 years.

Patient was on intravenous methylprednisolone 40 mg BD for last 12 days.

On examination, the patient was febrile (102.4 degrees Fahrenheit) with swelling in orbit and alveolar region of the face on left side.

**Table:** 

Ophthalmological examination	Right	Left
General condition of eye	Normal	Swelling, DOV, drooping
Vision with pin hole	6/36	PL negative
Vision unaided	6/36	PL negative
Non-contact tonometry	12 mm Hg	13 mm Hg
Ocular motility	Normal	Severely limited
Pupil	Poorly reacting	Mid-dilated/fixed
Slit-lamp	Unremarkable	Conjunctival chemosis
Fundoscopy	Unremarkable	Unremarkable

*Laboratory parameters* CRP: 28 mg/L; D-dimer: 855 ng/ml; serum ferritin: 470 ng/ml. CBC showed TLC of 13,000/mm^3^. Rest of the parameters (LFT, KFT, thyroid profile) were within normal limits.

**Fig. 8:**
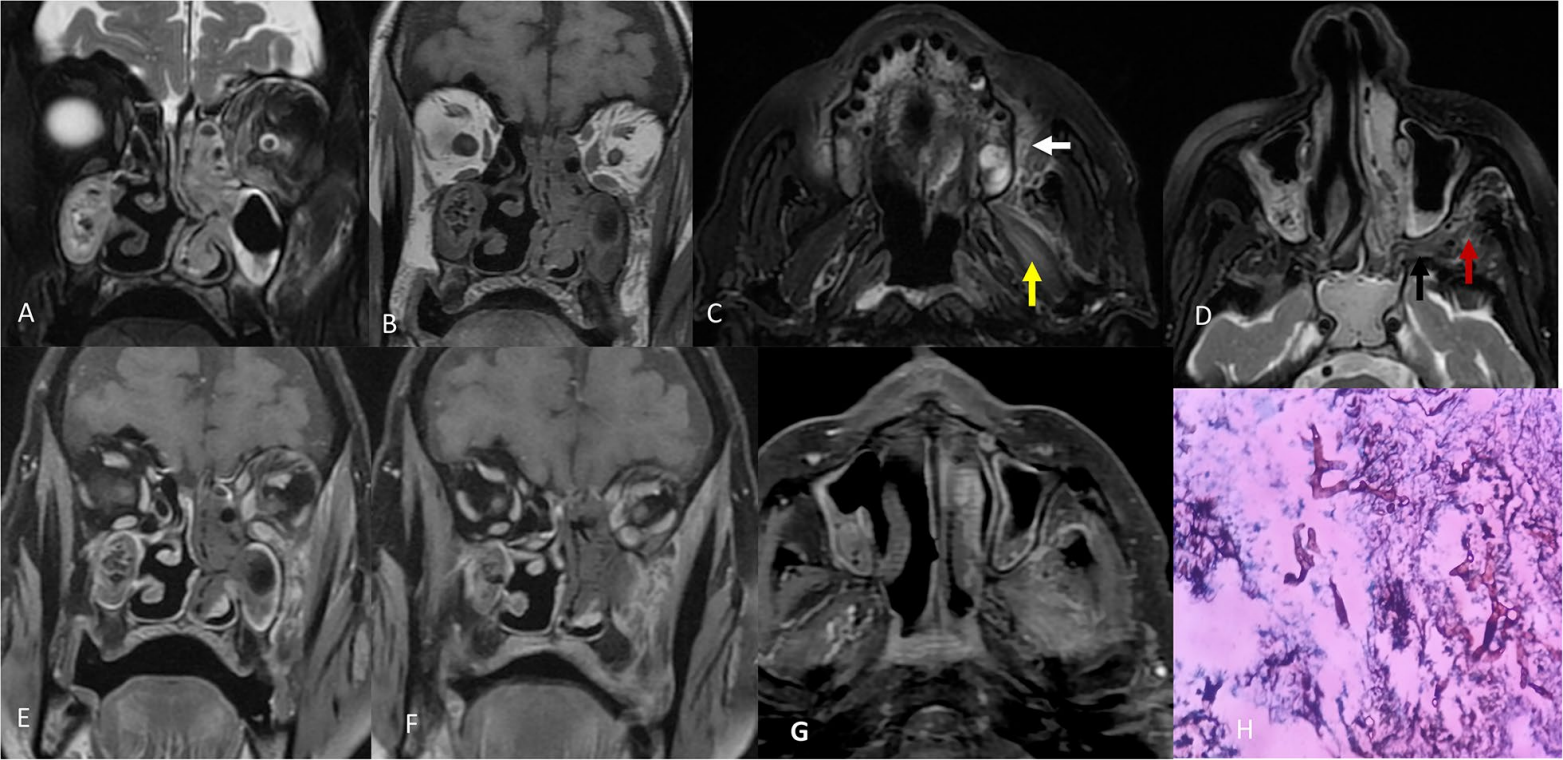
T2 fat suppressed coronal (**A**) and T1W coronal (**B**) images show evidence of extensive hypertrophy of turbinates on left side with opacification of bilateral maxillary and left ethmoidal sinuses with heterogeneous T2 hyperintense contents showing foci of signal void consistent with fungal elements. There is involvement of left orbit in contiguity with left ethmoid sinus in the form of bulky superior oblique, medial and inferior recti muscles with orbital fat stranding. Preseptal swelling and proptosis of left orbit were also present (not shown here). **C**, **D** T2W fat suppressed axial images showing complete opacification of sphenoid sinus in addition to above-mentioned findings along with hyperintensity in peri-alveolar (white arrow), pterygoid muscle (yellow arrow), pterygopalatine fossa (black arrow) and infratemporal fossa (red arrow) with corresponding enhancement on post contrast images (**E**–**G** T1 post contrast images). Post contrast images also show black turbinate sign. H: Microphotograph from debrided material showing black coloured aseptate fungal hyphae seen in the necrotic slough likely to be mucormycosis (GMS stain X400).

CEMRI of paranasal sinuses and orbits revealed evidence of rhino-orbital mucormycosis as shown in Fig. 8.

*Case 5* A 59-year-old female patient with uncontrolled diabetes mellitus and hypertension, was admitted with severe COVID-19. The patient was recovering well and was about to be discharged from hospital after a stay of 12 days. She received methylprednisolone 80 mg IV BD for 12 days which was to be tapered gradually. Now the patient complaints of fever, headache, altered consciousness and 2 episodes of seizures for the last 2 days.

**Fig. 9:**
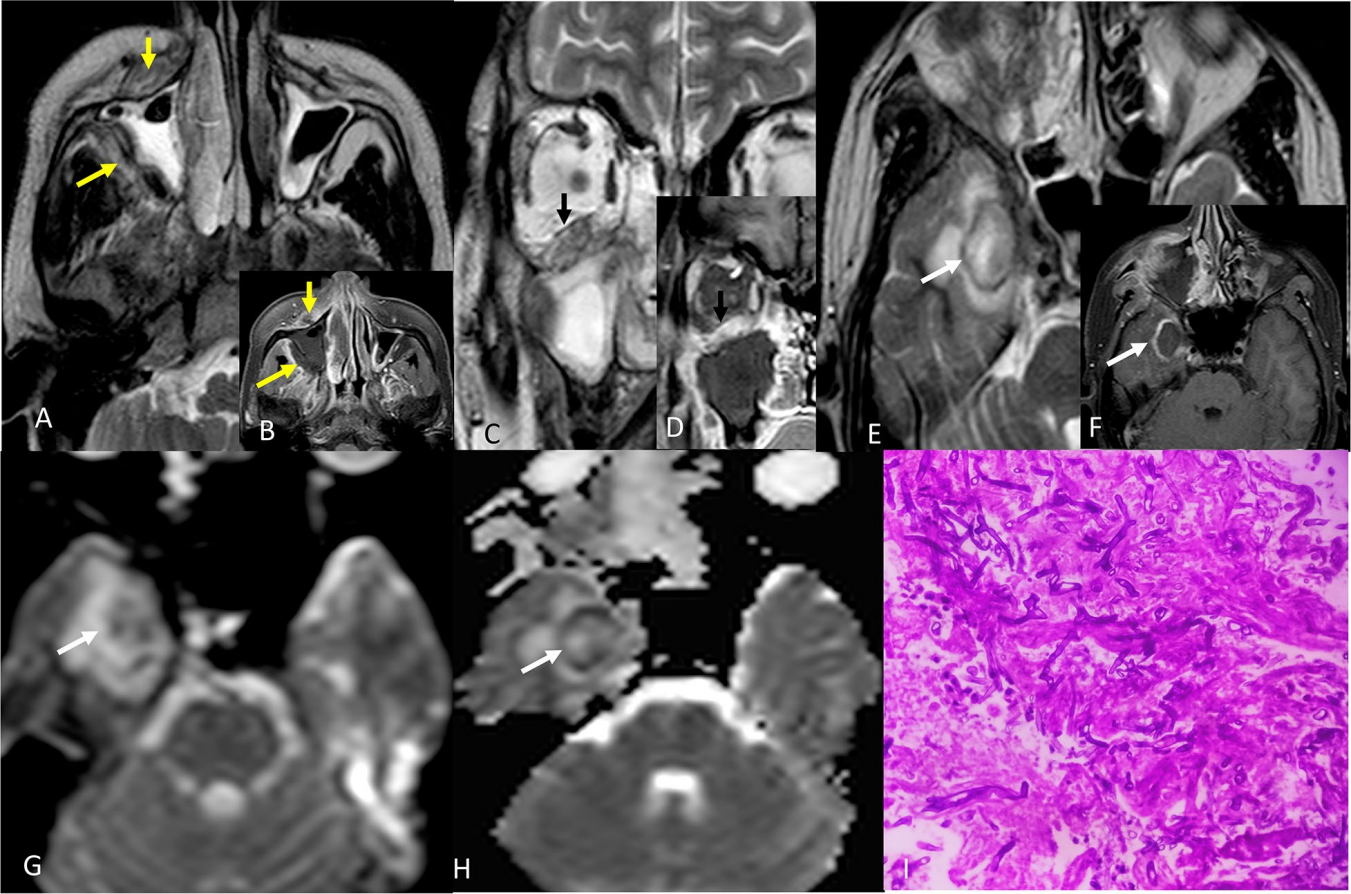
**A** (T2 axial) and **B** (T1 post contrast axial) images show mucosal thickening and opacification in bilateral maxillary sinuses with hypertrophy of turbinates on right side along with involvement of anterior and posterior periantral fat (yellow arrows) on right side with contiguous involvement of infratemporal fossa. **C** (T2 coronal), **D** (T1 post contrast coronal) images show involvement of inferior rectus muscle and fat stranding in right orbit. **E** (T2 axial), **F** (T1 post contrast axial) images of brain showing an abscess in right temporal lobe with hypointense wall on T2W image showing post contrast enhancement (white arrows). The wall of the abscess shows restriction of diffusion on DWI (**G**) with loss of signal intensity on ADC image (**H**). The internal contents do not show restriction of diffusion, suggesting fungal abscess. **I** Microphotograph from debrided material showing aseptate fungal hyphae seen in the necrotic material likely to be mucormycosis (H&E stain X400)

CEMRI of paranasal sinuses with orbits and brain revealed evidence of rhino-orbito-cerebral mucormycosis as shown in Fig. 9.

**Fig. 10:**
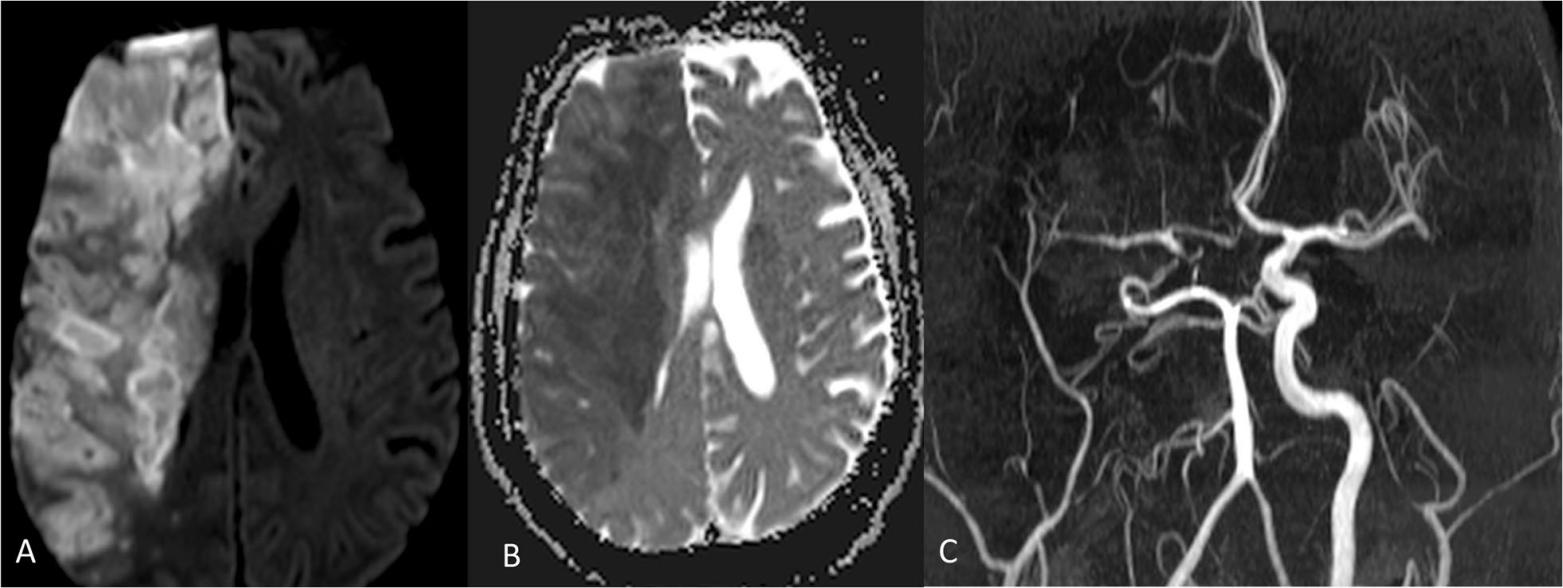
**A** (DWI) and **B** (ADC) images show acute infarct in right internal artery territory. **C** (MR angiography thick slab image): There is non-visualization of right internal carotid artery. Carotid Doppler (not shown) revealed a thrombus in proximal right internal carotid artery. Right middle cerebral artery is seen to be partly reformed by collaterals

*Case 6* A 46-year-old male, known diabetic and hypertensive, with moderate COVID-19 pneumonia presented with sudden onset of left hemiparesis. MRI brain (Fig. 10) revealed acute infarct with non-visualization of right internal carotid artery. Carotid doppler (not shown) revealed thrombus in proximal internal carotid artery.

## Conclusion

COVID-19 predominantly presents with respiratory symptoms and may cause a multitude of systemic complications involving various organs systems. One of the key components to understanding the pathogenesis of COVID-19 is downregulation of ACE-2, which is expressed on multiple organs and also counterbalances the pro-inflammatory ACE/angiotensin-II axis. This leads to influx of inflammatory cells into alveoli, increased vascular permeability and activation of prothrombotic mediators. We hypothesize the complications to be caused by either direct viral invasion or effect of cytokine storm leading to inflammation and thrombosis or a combination of both.

Imaging plays a key role in the early identification of COVID-19 related complications. Imaging findings such as ground glass opacities, interlobular septal thickening, vascular dilatation and pulmonary thrombosis correlate well with the pathogenesis described above. Rhino-orbital mucormycosis, a rare angio-invasive fungal infection has shown a rising trend in the setting of COVID-19, and requires prompt diagnosis and treatment to reduce the associated mortality and morbidity.

## Data Availability

The datasets used and/or analysed during the current study are available from the corresponding author on reasonable request.

## Abbreviations

COVID-19: Coronavirus disease 2019
SARS-CoV-2: Severe acute respiratory syndrome corona virus 2
WHO: World Health Organization
ACE-2: Angiotensin converting enzyme
CEMRI: Contrast enhanced magnetic resonance imaging
CT: Computed tomography
T1W: T1 weighted
T2W: T2 weighted
ARDS: Acute respiratory distress syndrome
RTPCR: Reverse transcriptase polymerase chain reaction
CRP: C reactive protein
TLC: Total leukocyte count
CBC: Complete blood count
LFT: Liver function test
KFT: Kidney function test
OD: Once a day
BD: Twice a day
BAL: Broncho-alveolar lavage
